# Peripheral leukocyte and endometrium molecular biomarkers of inflammation and oxidative stress are altered in peripartal dairy cows supplemented with Zn, Mn, and Cu from amino acid complexes and Co from Co glucoheptonate

**DOI:** 10.1186/s40104-017-0163-7

**Published:** 2017-05-01

**Authors:** Fernanda Batistel, Johan S. Osorio, Muhammad Rizwan Tariq, Cong Li, Jessica Caputo, Michael T. Socha, Juan J. Loor

**Affiliations:** 1 Department of Animal Sciences and Division of Nutritional Sciences, University of Illinois, 1207 West Gregory Drive, Urbana, IL 61801 USA; 2 Department of Dairy Science, South Dakota State University, Brookings, SD USA; 3 Department of Food Science and Technology, University College of Agriculture & Environmental Sciences, The Islamia University of Bahawalpur, Bahawalpur, Punjab Pakistan; 4 College of Animal Science and Technology, Key Laboratory of Animal Genetics and Breeding of Ministry of Agriculture, National Engineering Laboratory for Animal Breeding, China Agricultural University, Beijing, 100193 China; 5 Zinpro Corporation, Eden Prairie, MN USA

**Keywords:** Inflammation, Oxidative stress, Trace minerals, Transition period

## Abstract

**Background:**

Immune dysfunction and a higher risk of uterine infections are characteristics of the transition into lactation in dairy cows. The supply of complexed trace minerals, which are more bioavailable, could help overcome the greater needs of these nutrients in tissues around parturition and early lactation.

**Results:**

Twenty Holstein cows received an oral bolus with a mix of inorganic trace minerals (INO) or complexed trace minerals (AAC) to achieve 75, 65, 11, and 1 ppm supplemental Zn, Mn, Cu, and Co, respectively, in the total diet dry matter from -30 d through +30 d relative to parturition. Blood for polymorphonuclear leukocyte (PMNL) isolation was collected at -30, -15, +10, and + 30 d relative to parturition, whereas endometrium biopsies were performed at +14 and +30 d. Feeding AAC led to greater PMNL expression of genes related with inflammation response (*DDX58*), oxidative stress response (*MPO*), eicosanoid metabolism (*PLA2G4A* and *ALOX5AP*), transcription regulation (*PPARG*), and cellular adhesion (*TLN1*). The upregulation by AAC in endometrium of genes related with inflammation response (*TLR2, TLR4, NFKB1, TNF, IL6, IL1B, IL10, IL8*), prostaglandin synthesis (*PTGS2*, *PTGES*), and antioxidant responses (*NFE2L2*, *SOD1*) indicated a faster remodeling of uterine tissue and potentially greater capacity to control a local bacterial invasion.

**Conclusions:**

Data indicate that trace mineral supplementation from amino acid complexes improves PMNL activity and allows the prompt recovery of uterine tissue during early lactation. As such, the benefits of complexed trace minerals extend beyond an improvement of liver function and productive performance.

**Electronic supplementary material:**

The online version of this article (doi:10.1186/s40104-017-0163-7) contains supplementary material, which is available to authorized users.

## Background

The transition to lactation is a challenging period for dairy cows in large part because the immune system, e.g., neutrophil migration and phagocytosis, is generally dysfunctional [1–4]. Besides the hormonal and metabolic changes that contribute to a dysfunctional immune system, during parturition the physical barriers in the cervix, vagina and vulva also are compromised providing the opportunity for bacteria from the environment as well as the animal’s skin and feces to ascend the genital tract, hence, predisposing the cow to uterine diseases [5]. In the first 2 weeks after calving, 80–100% of cows present uterine colonization by bacteria, and an optimal response by the immune system is essential to rapidly eliminate the pathogens [5].

Neutrophils account for ca. 25% of leukocytes in bovine peripheral blood of healthy animals and they are the first line of innate immune defense against invading pathogens [2]. During uterine infection, toll-like receptors on endometrial cells recognize pathogen-associated molecules, leading to secretion of cytokines, antimicrobial peptides, and chemokines [6]. Chemokines recruit polymorphonuclear leukocytes (PMNL) into the site of infection within minutes and promote direct action against the microbes and attract lymphocytes; however, persistent infiltration is detrimental because the site of infection is continually exposed to pro-inflammatory cytokines and reactive oxygen metabolites (ROM) leading to chronic inflammation and oxidative stress and consequently subclinical endometritis and infertility [6].

Trace minerals are key components of antioxidant systems, metabolic reactions, protein synthesis pathways, and membrane integrity (physical barrier to pathogens) [4]. In postpartum dairy cows, supplementation of trace minerals (e.g., Zn, Se and Cu) benefits the immune system, and PMNL adhesion and superoxide production [7, 8]. While the demand of trace minerals increases around parturition, the blood and liver concentrations of trace minerals decreases [7, 9]. Thus, we hypothesized that supplementation of trace minerals through more bioavailable forms, e.g., amino acid complexes, would benefit recovery of the endometrium and the innate immune response at least in part by altering the expression of genes associated with PMNL activity and inflammation. Therefore, the objective of the present study was to evaluate the effects of organic trace mineral supplementation on expression of key genes associated with inflammation, oxidative stress, and eicosanoids in PMNL and endometrium tissue. Production responses and biomarkers of energy balance have been reported elsewhere [10].

## Methods

All the procedures for this study were conducted in accordance with the protocol approved by the Institutional Animal Care and Use Committee (IACUC) of the University of Illinois (Protocol #12097).

### Animals, experimental design, and dietary treatments

Details of the experiment design have been published previously [10]. Briefly, 44 multiparous Holstein cows were blocked (6 cows per block) according to parity, previous lactation milk yield, and expected day of parturition. All cows received a common diet from -110 to -30 d relative to parturition and were supplemented at 100% of the National Research Council [11] requirements with Zn, Mn, Cu, and Co in the form of an inorganic trace mineral mix (INO). From -30 d relative to expected day of parturition, cows received a common prepartal diet (close-up diet), and from calving to 30 d in milk (DIM) a common postpartal diet (fresh diet). Both close-up and fresh diet were partially supplemented with an INO mix of Zn, Mn, and Cu to supply 35, 45, and 6 ppm, respectively, of the total dietary minerals. The diets and chemical composition are presented in Table 1. At -30 d relative to parturition, cows were randomly assigned to an oral administration of a bolus once daily at the time of feeding the TMR. This contained a mix of either inorganic (INO) or complexed (AAC) Zn, Mn, Cu, and Co to achieve 75, 65, 11, and 1 ppm supplemental, respectively, in the total diet dry matter intake (DMI). The complexed trace minerals were provided as Availa®Zn (Zn AA complex), Availa®Mn (Mn AA complex), Availa®Cu (Cu AA complex), and CoPro® (Co glucoheptonate) (Zinpro Corp, Eden Prairie, MN) and the inorganic trace minerals in sulfate form. IACUC approved uterine biopsies in a maximum of 12 cows per group, which was deemed appropriate to detect statistical significance based on previous research [12–14]. However, only 20 (AAC = 9; INO = 11) out of 44 cows used for this study had a complete set of uterine endometrial biopsies and PMNL isolations. Per IACUC guidelines, cows with a clinical disorder could not continue on experiment; thus, a total of 7 cows had to be removed from the experiment due to clinical ketosis, clinical mastitis, retained placenta, displaced abomasum, or leg fracture [10]. All cows used for PMNL and endometrium gene expression were clinically-healthy.

**Table 1:** Ingredient and analyzed chemical composition of diets fed during close-up (-30 d to calving) and early lactation (1 to 30 d in milk)

Component^a^	Far-off	Close-up	Early lactation
Ingredient, % of DM			
Alfalfa silage	12.2	7.6	4.9
Alfalfa hay	-	3.5	3.9
Corn silage	33.6	38.9	33.1
Wheat straw	34.8	8.4	2.6
Cottonseed	-	-	3.9
Wet brewers grains	-	6.1	9.4
Ground shelled corn	4.9	18.8	22.6
Soy hulls	2.0	4.1	3.9
Soybean meal, 48% CP	8.9	3.0	5.6
Expeller soybean meal^b^	-	0.7	0.2
SoyChlor^c^	0.2	2.3	-
Blood meal 85% CP	1.0	0.6	0.3
Molasses	-	0.4	-
Urea	0.3	-	0.7
Rumen-inert fat^d^	-	-	2.0
Limestone	0.8	2.2	1.6
Salt (plain)	0.3	-	0.3
Ammonium chloride	-	1.14	-
Dicalcium phosphate	0.1	0.3	0.4
Magnesium oxide	-	0.1	0.1
Magnesium sulfate	0.2	1.4	0.3
Sodium bicarbonate	-	-	0.7
Calcium sulfate	-	-	0.1
Mineral-vitamin mix^e^	0.2	0.2	0.2
Vitamin A^f^	0.02	0.03	0.04
Vitamin D^g^	0.01	0.02	0.02
Vitamin E^h^	0.36	0.36	0.20
Chemical analysis			
NE_L_, Mcal/kg DM	1.25	1.59	1.67
CP, % DM	14.4	14.3	18.7
NDF, % DM	53.0	39.1	35.9
ADF, % DM	34.5	23.9	22.2
Zn, mg/kg of DM	103	83	69
Mn, mg/kg of DM	84	76	70
Cu, mg/kg of DM	15.5	14.4	12.3
CO, mg/kg of DM	0.83	0.72	0.19

^a^Basal close up and lactation diets were considered as basal diet plus

inorganic trace minerals, or basal diet plus organic trace minerals

^b^SoyPLUS (West Central Soy, Ralston, IA)

^c^SoyChlor (West Central Soy)

^d^Energy Booster 100 (MSC, Carpentersville, IL)

^e^Contained a minimum of 4.3% Mg, 8% S, 6.1% K, 2.0% Fe, 3.0% Zn,

3.0% Mn, 5,000 mg/kg of Cu, 250 mg/kg of I, 40 mg/kg of Co, 150

mg/kg of Se, 2,200 kIU/kg of vitamin A, 660 kIU/kg of vitamin D_3_,

and 7,700 IU/kg of vitamin E

^f^Contained 30,000 kIU/kg

^g^Contained 5,009 kIU/kg

^h^Contained 44,000 IU/kg

### Sample collection

Blood samples (120 mL) were collected from the tail vein using 20-gauge BD Vacutainer needles (Becton Dickinson, Franklin Lakes, NJ) and vacutainers (8 mL, Becton Dickinson, Franklin Lakes, NJ) containing solution A of trisodium citrate, citric acid and dextrose (ACD) at -30, -15, +10 and +30 d relative to parturition. After blood collection, the tubes were mixed well by inversion and placed on ice until PMNL isolation (~30 min).

Endometrial biopsies were collected by a single individual at +14 and +30 d relative to calving following similar procedures described previously [15]. Briefly, an epidural was performed (4 mL of 2% lidocaine) prior to introducing a Hauptner biopsy instrument protected with a sanitary chemise into the vagina. Manipulation per rectum allowed the biopsy tool to pass through the cervix, after which the biopsy instrument alone was introduced into the uterus subsequent to rupturing the sanitary chemise at the external cervical orifice. The tool was guided into the uterine horn approximately 5 cm past the uterine bifurcation. The tip of the biopsy instrument inside the uterus was carefully identified using the non-operating hand per rectum. This approach should have allowed the reproducible procurement of tissue. With the help of the hand in the rectum, the medial uterine wall was gently pressed into the open instrument jaws prior to closing the jaws and withdrawing the instrument. No attempt was made to determine the relative contribution of caruncular and non-caruncular tissue in the biopsies, even though there is some evidence for differences in transcriptome profiles [16]. The tissue clipped off was immediately placed in liquid nitrogen and frozen at -80 °C until RNA extraction.

### Polymorphonuclear leukocyte (PMNL) isolation and viability analysis

Complete details of PMNL isolation and viability analysis are included in the Additional file 1. Briefly, PMNL were isolated from whole blood collected in ACD-containing vacutainers. An aliquot (20 μL) obtained during the isolation process was used for PMNL quantification and viability using a granulocyte primary antibody (CH138A, Veterinary Microbiology and Pathology, Washington State University, Pullman, WA) followed by a second antibody (Goat Anti-Mouse IgM, Human ads-PE, Southern Biotech, Birmingham, AL). Cells were fixed with 150 μL of 4% paraformaldehyde (Sigma-Aldrich, St. Louis, MO) and preserved at 4 °C until flow cytometry reading (LSR II, Becton Dickinson, San Jose, CA). All samples harvested and used for analysis contained more than 80% PMNL and had at least 90% viability.

### RNA extraction, primer design and evaluation, and quantitative PCR

Methods for RNA extraction from PMNL and endometrium, primer design and evaluation, cDNA synthesis, quantitative reverse transcription PCR and gene function are presented in the Additional files 2, 3 and 4. Briefly, RNA samples were extracted using Qiazol reagent in combination with the miRNeasy® Mini Kit (Cat. #217004, Qiagen). Thirty-two target genes involved in inflammation response, oxidative stress, eicosanoid metabolism, cellular receptors, transcription regulation and glucose metabolism were evaluated in the PMNL, while 30 target genes related to inflammation, oxidative stress, eicosanoid metabolism, transcription regulation and antimicrobial peptides were assessed in the endometrium. Primers were designed via Primer Express 3.0.1 software (Applied Biosystems). Quantitative PCR (qPCR) was performed in an ABI Prism 7900 HT SDS instrument (Applied Biosystems). Details of primer sequences and amplicon size, primer product sequencing information, and qPCR performance are presented in the Additional file 5, 6, 7 and 8. For PMNL, the internal controls were *GOLGA5*, *SMUG1*, and *OSBPL2* [17, 18], while for endometrium were *GAPDH*, *RPS9*, and *UXT*. The geometric mean of the internal control genes was used to normalize the expression data.

### Statistical analysis

Data were analyzed using the MIXED procedure of SAS 9.3 (SAS Institute Inc., Cary, NC) according to the following model:

$$ {Y}_{i jkl}\kern0.5em =\kern0.75em \upmu +{D}_i+{b}_j+{c}_k+{T}_l + D{T}_{i l} + {e}_{i jkl} $$

Where *Y*_*ijkl*_ represent the dependent variable; μ is the overall mean; *D*_*i*_ is the fixed effect of treatment (i = 1, 2); *b*_*j*_ is the random effect of block (j = 1, …9); *c*_*k*_ is the random effect of cow within treatment and block (l = 1…, n_*ij*_); *T*_*l*_ is the fixed effect of time (day or week) of the experiment (m = 1,… n); *DT*_*il*_ is the fixed effect of treatment by time interaction; and *e*_*ijkl*_ is the residual error. Endometrium gene expression results were log_2_-scale transformed in order to comply with normal distribution of residuals. For PMNL, the gene expression data at -15, +10, and +30 d relative to parturition was expressed as fold-change relative to -30 d. Statistical differences were declared significant at *P* ≤ 0.05 and tendencies at *P* ≤ 0.10.

## Results

### PMNL

#### Inflammation response

**Fig. 1:**
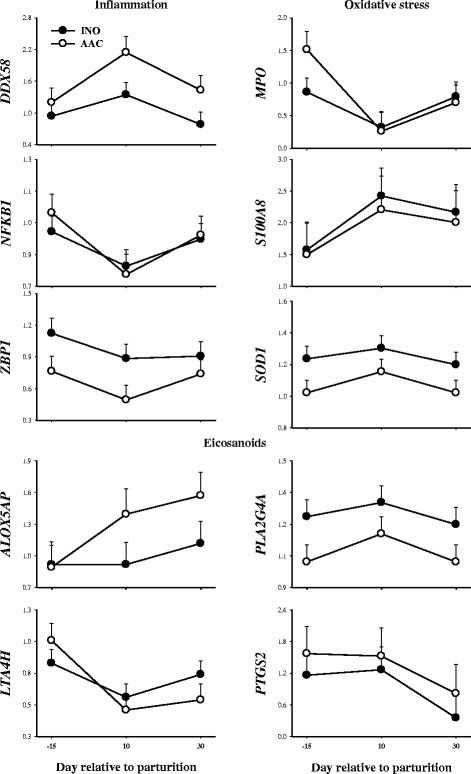
mRNA expression (fold-change relative to -30 d prepartum) of genes associated with inflammation (*DDX58, NFKB1* and *ZBP1*), oxidative stress (*MPO, S100A8* and *SOD1*) and eicosanoids (*ALOX5AP*, *LTA4H, PLA2G4A* and *PTGS2*) in polymorphonuclear leukocytes (PMNL) of cows supplemented with inorganic (INO; *n* = 11) or complexed (AAC; *n* = 9) trace minerals during the pre- and postpartal period

The cell surface receptors *TLR2* (*P =* 0.85) and *TLR4* (*P =* 0.48), which are involved in the inflammation-response were not affected by treatments (Table 1). The transcription factors *STAT3* (*P =* 0.62), *TNF* (*P =* 0.14) and *NFKB1* (*P =* 0.75) also were not affected by treatments. Among the proteins that recognize foreign DNA, *DDX58* had greater expression (*P* = 0.05; Table 2 and Fig. 1) in the AAC compared with INO cows, while there was a tendency (*P* = 0.09) for the opposite effect for *ZBP1*; however, *IPS1* (*P =* 0.23) was not affected by treatments (Table 2). There was an overall decrease in expression of *NFKB1* (*P =* 0.03) from -15 to +10 d regardless of treatment (Fig. 1).

**Table 2:** Effects of supplementing cows with inorganic (INO, *n* = 11) or complexed (AAC, *n* = 9) trace minerals during the peripartal period on mRNA expression (fold-change relative to -30 d prepartum) of genes related with inflammation response, oxidative stress, eicosanoids, transcription factors, receptors and glucose metabolism in polymorphonuclear leukocytes (PMNL)

Gene	Treatments		SEM^a^	*P* value^1^		
	INO	AAC		Treatment	Time	T × T^b^
Inflammation						
*DDX58*	1.02	1.59	0.21	0.05	0.01	0.40
*IPS1*	0.80	1.00	0.13	0.23	0.41	0.22
*NFKB1*	0.89	0.91	0.06	0.75	0.03	0.74
*STAT3*	1.17	1.09	0.12	0.62	0.62	0.84
*TLR2*	1.27	1.21	0.25	0.85	0.84	0.41
*TLR4*	1.14	1.43	0.30	0.48	0.11	0.49
*TNF*	1.33	0.82	0.25	0.14	0.99	0.68
*ZBP1*	0.97	0.66	0.13	0.09	0.15	0.62
Oxidative stress						
*MPO*	0.66	0.83	0.18	0.48	<0.01	0.03
*NFE2L2*	1.32	1.57	0.30	0.54	0.15	0.70
*NOX1*	0.97	0.99	0.26	0.94	0.61	0.12
*S100A8*	2.05	1.90	0.40	0.78	0.08	0.98
*SOD1*	0.73	0.91	0.09	0.14	0.02	0.94
*SOD2*	1.38	1.72	0.22	0.27	0.70	0.99
*SOD3*	0.66	0.06	0.31	0.15	0.32	0.70
Eicosanoids						
*ALOX5AP*	0.98	1.28	0.12	0.09	0.10	0.35
*LTA4H*	0.71	0.67	0.10	0.76	<0.01	0.18
*PLA2G4A*	0.84	1.23	0.15	0.06	0.22	0.23
*PTGS2*	0.93	1.30	0.46	0.53	0.04	0.93
Transcription factors						
*PPARA*	1.03	0.63	0.14	0.04	0.01	0.21
*PPARD*	1.14	1.23	0.16	0.71	0.30	0.74
*PPARG*	0.84	1.68	0.33	0.09	0.80	0.79
*RXRA*	1.21	1.03	0.11	0.22	0.05	0.78
Receptors						
*ADORA1*	1.08	1.55	0.14	0.01	0.08	0.15
*ENTPD1*	1.14	1.64	0.23	0.12	0.34	0.80
*IL10*	1.37	1.36	0.20	0.95	0.16	0.85
*IL1B*	1.22	1.76	0.45	0.38	0.51	0.77
*ITGAM*	0.75	0.78	0.07	0.74	0.10	0.32
*ITGB2*	0.99	0.86	0.08	0.22	0.34	0.29
*P2RY11*	1.02	0.89	0.18	0.60	0.14	0.44
*PANX1*	0.86	0.95	0.13	0.61	0.02	0.15
*SELL*	1.41	1.66	0.41	0.65	0.27	0.80
*TLN1*	0.93	1.04	0.03	0.01	0.60	0.74
*VCL*	0.74	0.83	0.06	0.25	<0.01	0.76
Glucose metabolism						
*LDHA*	1.37	0.84	0.20	0.06	0.04	0.95
*SLC2A1*	0.83	0.56	0.06	<0.01	<0.01	<0.01

^1^ *P* values represents the probability of statistical significance for the fixed effects (treatment, time, treatment × time). Statistical differences were declared significant at *P* ≤ 0.05 and tendencies at *P* ≤ 0.10

^a^Largest standard error of the mean is shown

^b^Interaction of treatment × time

### Oxidative stress

An interaction T × T (treatment × time; *P* = 0.03) was detected for *MPO* due to its upregulation at -15 d in the AAC cows (Table 2, Fig. 1). The expression of the superoxide dismutase enzymes *SOD1* (*P =* 0.14), *SOD2* (*P =* 0.27) and *SOD3* (*P =* 0.15) was not affected by treatment. However, the expression of *SOD1* increased from -15 to +10 d regardless of treatment (Table 2, Fig. 1). The oxidant scavenger proteins *S100A8* (*P =* 0.78) and *NOX1* (*P =* 0.94) as well as the transcription factor *NFE2L2* (*P =* 0.54) were not affected by treatments (Table 2, Fig. 1). However, *S100A8* was upregulated (*P =* 0.08) from -15 to +10 d in both treatments.

### Eicosanoids

Among the genes related with arachidonic acid, *PLA2G4A* (*P* = 0.06) and *ALOX5AP* (*P* = 0.09) tended to have greater expression in cows fed AAC (Table 2, Fig. 1). In contrast, the mRNA expression of *LTA4H* (*P =* 0.76) and *PTGS2* (*P =* 0.53) were not affected by treatments (Table 2, Fig. 1). A marked decrease (*P* < 0.01) in expression of *LTA4H* was observed between -15 and +10 d regardless of treatment, whereas *PTGS2* expression gradually decreased (*P* = 0.04) between -15 and +30 d regardless of treatment (Fig. 1).

### Transcription factors

**Fig. 2:**
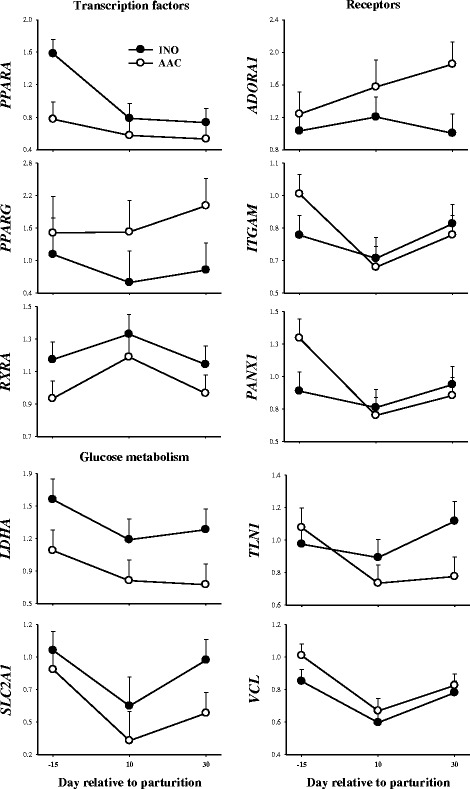
mRNA expression (fold-change relative to -30 d prepartum) of genes associated with transcription factors (*PPARA, PPARG* and *RXRA*), receptors (*ADORA1, ITGAM, PANX1*, *TLN1* and *VCL*) and glucose metabolism (*LDHA* and *SLC1A1*) in polymorphonuclear leukocytes (PMNL) of cows supplemented with inorganic (INO; *n* = 11) or complexed (AAC; *n* = 9) trace minerals during the pre- and postpartal period

*PPARA* had lower overall mRNA expression (*P* = 0.04) in AAC cows, whereas *PPARG* tended to have greater mRNA expression compared with INO (*P* = 0.09; Table 2 and Fig. 2). The mRNA expression of *PPARD* (*P =* 0.71) and *RXRA* (*P =* 0.22) were not affected by treatments (Table 2). However, expression of *RXRA* increased (*P* = 0.05) from -15 to +10 d and then decreased to prepartum values in both treatments (Fig. 2).

### Receptors

The expression of the receptors *TLN1* (*P* = 0.01) and *ADORA1* (*P* = 0.01) was greater in the cows receiving AAC treatment (Table 2, Fig. 2). However, the treatments did not alter (all *P* > 0.10) expression of other receptors measured (*SELL, ITGAM, ITGB2, VCL, PANX1, ENTPD1, P2RY11, IL1B,* and *IL10*) (Table 2, Fig. 2)*.*

### Glucose metabolism

An interaction T × T (*P* < 0.01) was detected for the glucose transporter *SLC2A1* due to the marked decrease in expression between -15 and +10 d. Whereas, the mRNA expression of *LDHA* (*P* = 0.06) tended to be lower in the AAC compared with INO cows (Table 2, Fig. 2).

### Endometrium

#### Inflammation response

**Fig. 3:**
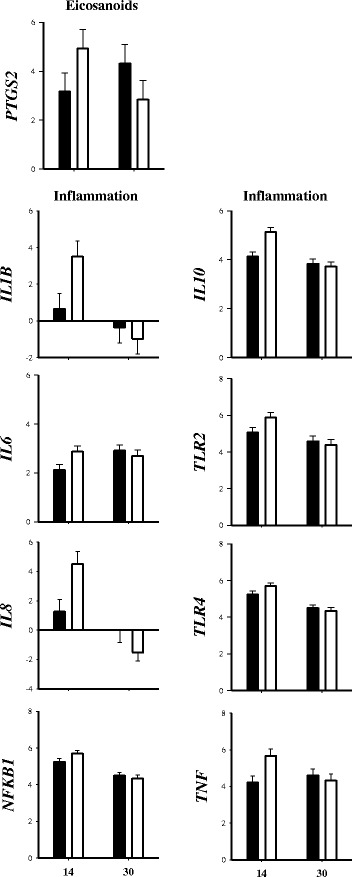
mRNA expression (log 2-scale) of genes associated with immune-related receptors (*TLR2* and *TLR4*), pro-inflammatory response (*NFKB1, TNF, IL6, IL1B* and *IL8*), anti-inflammatory response (*IL10*) and eicosanoids (*PTGS2*) in endometrium of cows supplemented with inorganic (INO; *n* = 11) or complexed (AAC; *n* = 9) trace minerals during the pre- and postpartal period

A tendency for a T × T interaction was detected for *TLR2* (*P* = 0.08), *TLR4* (*P* = 0.08), *TNF* (*P* = 0.10), and *NFKB1* (*P* = 0.06) due to greater mRNA expression in AAC cows at +14 d, whereas lower expression was observed at +30 d (Table 3, Fig. 3). Furthermore, a T × T was observed for *IL6* (*P* = 0.03), *IL1B* (*P* < 0.01), *IL8* (*P* < 0.01), and *IL10* (*P* < 0.01) because all these genes had greater mRNA expression in the AAC cows at +14 d but no treatment effect was detected at +30 d. In addition, AAC treatment increased *STAT3* (*P* = 0.05) and tended to increase *MYD88* (*P* = 0.06) mRNA expression (Table 3). The expression of *MYD88* decreased (*P* = 0.05) regardless of treatment from +14 to +30 d postpartum.

**Table 3:** Effects of supplementing cows with inorganic (INO; *n* = 11) or complexed (AAC; *n* = 9) trace minerals during the peripartal period on mRNA expression (log-2 scale) of genes related with inflammation response, oxidative stress, eicosanoids, transcription factors and antimicrobial peptides in endometrium tissue at +14 and +30 d after parturition

Gene	Treatments				SEM^a^	*P* value^1^		
	INO		AAC					
	+14	+30	+14	+30		Treatment	Time	T × T^b^
Inflammation								
*IL10*	4.13	3.83	5.13	3.72	0.19	0.04	<0.01	<0.01
*IL1B*	0.65	-0.37	3.51	-0.98	0.84	0.26	<0.01	<0.01
*IL6*	2.12	2.91	2.88	2.70	0.23	0.24	0.15	0.03
*IL8*	1.26	-0.01	4.51	-1.52	0.84	0.37	<0.01	<0.01
*MYD88*	4.98	4.86	5.46	4.93	0.15	0.06	0.05	0.20
*NFKB1*	5.06	5.36	5.41	5.06	0.16	0.87	0.90	0.06
*SAA3*	3.10	2.77	4.88	2.06	1.00	0.46	0.15	0.25
*STAT3*	5.05	5.22	5.69	5.16	0.19	0.05	0.40	0.11
*TLR2*	5.05	4.58	5.87	4.39	0.28	0.19	<0.01	0.10
*TLR4*	5.24	4.49	5.69	3.34	0.18	0.40	<0.01	0.08
*TNF*	4.21	4.60	5.67	4.32	0.36	0.11	0.12	0.01
Oxidative stress								
*NFE2L2*	4.85	5.11	5.20	5.26	0.13	0.06	0.22	0.43
*NOS3*	4.94	5.16	5.11	5.07	0.36	0.91	0.76	0.66
*NRROS*	5.18	4.62	5.20	4.87	0.32	0.68	0.13	0.70
*SOD1*	5.19	5.04	5.26	5.28	0.09	0.09	0.39	0.30
*SOD2*	4.83	4.13	5.57	3.83	0.32	0.44	<0.01	0.12
*SOD3*	4.87	5.27	5.08	5.16	0.26	0.85	0.28	0.45
Eicosanoid synthesis								
*ALOX5*	4.89	4.83	5.08	5.14	0.41	0.51	0.99	0.88
*ALOX5AP*	4.86	4.92	5.33	4.95	0.38	0.51	0.60	0.48
*LTA4H*	5.07	5.30	5.24	5.23	0.10	0.51	0.30	0.24
*LTC4S*	3.91	3.87	4.35	3.98	0.42	0.45	0.62	0.70
*PLA2G4A*	4.92	5.39	5.44	4.96	0.29	0.86	0.99	0.13
*PTGDS*	5.26	4.94	5.05	4.87	0.29	0.63	0.34	0.77
*PTGES*	4.50	3.68	5.53	3.92	0.34	0.10	<0.01	0.15
*PTGS2*	3.17	4.32	4.94	2.85	0.77	0.83	0.52	0.04
Transcription regulation								
*PPARA*	5.09	5.48	5.20	5.68	0.21	0.48	0.02	0.79
*PPARD*	5.47	5.13	5.46	5.07	0.15	0.81	0.01	0.89
*PPARG*	4.72	5.17	4.64	6.03	0.31	0.21	<0.01	0.11
*RXRA*	5.28	5.24	5.24	5.09	0.13	0.49	0.38	0.60
Antimicrobial peptides								
*MUC1*	2.84	3.78	4.29	4.05	0.46	0.04	0.49	0.25

^1^ *P* values represents the probability of statistical significance for the fixed effects (treatment, time, treatment × time). Statistical differences were declared significant at *P* ≤ 0.05 and tendencies at *P* ≤ 0.10

^a^Largest standard error of the mean is shown

^b^Interaction of treatment × time

### Oxidative stress

No T × T was observed (*P* > 0.10) for genes related with oxidative stress (Table 3). However, mRNA expression of *SOD1* (*P* = 0.09) and *NEF2L2* (*P* = 0.06) tended to be greater in AAC compared with INO cows (Table 3). In addition, expression of *SOD2* decreased (*P* < 0.01) regardless of treatment from +14 to +30 d postpartum.

### Eicosanoids

A T × T (*P* = 0.04) was observed for *PTGS2* because its expression became greater over time with INO while it decreased with AAC (Table 3, Fig. 3). A tendency (*P* = 0.10) for a greater overall *PTGES* mRNA expression was observed for AAC cows (Table 3). In addition, expression of *PTGES* decreased (*P* < 0.01) from +14 to +30 d postpartum regardless of treatment (Table 3).

### Transcription factors

No T × T or overall treatment effect (*P* > 0.10) was observed for *PPARA, PPARD, PPARG* and *RXRA*. However, expression of *PPARA* and *PPARG* increased and *PPARD* decreased from +14 to +30 d postpartum (Table 3).

### Antimicrobial peptides

*MUC1* mRNA expression was upregulated (*P* = 0.04) overall in the AAC compared with INO cows (Table 3).

## Discussion

### Neutrophil function around calving

Neutrophil function and bactericidal efficiency is compromised during the transition period [1]. A lower capacity for trafficking, phagocytosis, and pathogen killing during this period is partly associated with changes in hormones and metabolites and with immune or stress-like conditions [19]. Some studies also have reported that neutrophils have impaired generation of ROM during the transition period [20, 21], a feature that may contribute to greater susceptibility to disorders in early lactation. The general hypothesis of this study was that feeding trace minerals through more bioavailable forms would improve immune health, enhance PMNL activity, and help overcome the challenges of the transition period [10]. In the companion paper [10] we reported production and biomarker data indicating that better DMI and milk production in cows fed AAC was partly due to better liver function and PMNL phagocytosis.

### Inflammation response

The activation of innate immune responses are regulated by several DNA sensors including toll-like receptors, e.g., TLR2, TLR4, DDX58 and ZBP1 [22]. Subsequent to pathogen detection, these receptors activate signaling pathways (e.g., STAT3 and NF-κB), which trigger the synthesis of pro-inflammatory cytokines and chemokines [23]. Despite the fact that cows in the AAC treatment had greater expression of *DDX58* and lower expression of *ZBP1*, those responses did not seem to activate the pro-inflammatory pathways as indicated by the lack of change in expression of the cytokines *TNF* and *IL1B*.

### Oxidative stress

Myeloperoxidase (*MPO*) is the main peroxidase enzyme released upon neutrophil activation, and catalyzes the formation of hypochlorous acid, a potent oxidant that displays bactericidal activity [24]. Furthermore, MPO has traditional cytokine-like function and acts as an autocrine modulator of neutrophil activation [25]. In steers, a Cu-deficient diet impaired neutrophil killing capacity without altering phagocytosis [26]. Similarly, Cu-depleted calves exhibited impaired phagocytic killing activity, which was restored by Cu supplementation [27]. Therefore, the greater mRNA expression of *MPO* indicated that AAC cows were more likely to have greater PMNL activation, hence, superior capacity to kill invading pathogens.

When ROM production elicits a metabolic imbalance in cells, the release of endogenous neutralizing agents helps to minimize their potential deleterious effects. The protein S100A8 comprises ~20% of the PMN cytoplasm [28], and exerts an important protective mechanism during inflammation because it scavenges intracellular ROM produced by activated PMN and attenuates nitric oxide production [29]. Similarly, the cytoplasmic enzyme SOD1 transforms the harmful superoxide radicals to molecular oxygen and hydrogen peroxide [30]. Therefore, the tendency for upregulation of *SOD1* and *S100A8* at +10 d regardless of treatment could be taken as an indication of PMNL attempting to neutralize the greater oxidative stress experienced after parturition [31].

### Eicosanoid metabolism

Neutrophil stimulation produces oxygen-derived reactive species, lysosomal enzymes, nitric oxide as well as pro-inflammatory and anti-inflammatory mediators which include bioactive lipids such as the eicosanoids (e.g., prostaglandins and leukotrienes) [32]. The tendency for upregulation of *PLA2G4A* in AAC cows could have resulted in an increase in the hydrolysis of cell membrane phospholipids to release arachidonic acid, which subsequently could be used for leukotriene (via *ALOX5AP*) synthesis. Leukotrienes, such as leukotriene B4, are essential components of the inflammatory response because they act as chemoattractants for mature neutrophils, and promote neutrophil activation [33]. Furthermore, leukotriene B4 enhances cytokine production and the presence of these fatty acids seems to determine the duration and magnitude of the inflammatory response [34]. In vitro, Zn, Cu, and Ni enhanced PMN motility by chemotactic activation indicating that the inflammatory response can be partly modulated through the availability of those metals [35].

### Transcription regulation

In non-ruminants, the family of transcription factors termed peroxisome proliferator-activated receptors (PPAR) is involved in the control of inflammation [34]. Eicosanoids are PPARα activators [36] that can inhibit arachidonic acid-induced inflammation in part by enhancing degradation of leukotriene B4 [37]. It is noteworthy that the expression of *ALOX5AP* (leukotriene synthesis) and *PPARA* followed opposite patterns of expression with the AAC treatment indicating that the inflammatory response in those cows likely was of a greater magnitude but of brief duration. The absence of change in the expression of the pro-inflammatory cytokine *IL1B* may be due to the upregulation of *PPARG* in the AAC treatment. Prior research in non-ruminant cells indicated an inhibitory effect of PPARγ on cytokine production [34].

### Receptors

Neutrophil recruitment and migration to inflamed tissues are critical for proper immune function. Cytoskeletal proteins, such as talin-1 (*TLN1*)*,* facilitate the transition from neutrophil rolling to arrest [38]. Furthermore, modulation of neutrophil function by adenosine (*ADORA1*) promotes neutrophil chemotaxis and phagocytosis [39]. Therefore, the upregulation of *TLN1* and *ADORA1* in AAC cows indicated that PMNL were better equipped to be deployed into the inflamed sites. Despite these unique effects of AAC, the overall downregulation of *PANX1* and *VCL* from -15 to +10 d regardless of treatment seemed to indicate a degree of impairment in the recruitment of PMNL and their ability to adhere to endothelium. Some evidence indicates that *PANX1* channels are activated by ATP [40], which may explain the gradual upregulation of *ADORA1* over time, i.e., a counter regulatory mechanism to help regulate PMNL activity. In addition, the downregulation of *VCL* could partly be explained by gradual degradation of PMNL plasma membrane phosphatidylinositol 4,5-bisphosphate, which is essential for activation of *VCL* [41]. Whether such effect is directly related to catabolic enzymes (e.g., phospholipases) or greater turnover of PMNL is unknown.

### Glucose metabolism

At least in non-ruminants, neutrophils rely on glycolysis as the main source of energy; however, the extra energy required for phagocytosis is usually derived from metabolism of lactate [42]. Both *SCL2A1* and *LDHA* are important regulators of energy metabolism in neutrophils. The first facilitates the transport of glucose across the plasma membrane, whereas the second is involved in the interconversion of pyruvate to lactate after glycolysis [43]. The parallel downregulation of *SLC2A1* and *LDHA* regardless of treatment to a nadir at +10 d postpartum was most likely a result of the shortfall in circulating glucose commonly observed after parturition [10]. The numerically-greater expression of *SLC2A1* and *LDHA* in INO cows during the study could indicate that these cows were more immuno-compromised because a previous study detected marked upregulation of *LDHA* in PMNL after a mastitis challenge [14]. If such an effect existed it could help to partly explain the lower phagocytic activity in whole blood that was measured on +30 d in INO cows [10]. Because cows in INO had greater plasma concentrations of ketones, the upregulation of *LDHA* in these cows could have been a mechanism induced by ketone body (e.g., hydroxybutyrate) metabolism to decrease glucose oxidation by the PMNL [44].

### Endometrium

Bacterial contamination and consequent inflammatory response of the uterine tissue after parturition are common and are associated with lower conception rates, longer interval periods from calving to first service or conception, and more animals culled for failure to conceive [45]. Considering that trace minerals play important roles in the health and immunity of peripartal dairy cows [46] and complexed trace mineral supplementation in partial substitution of sulfate sources elicited an improvement in immune function [10, 47], it was important to ascertain if complexed trace minerals also elicited a local response in the endometrium.

### Inflammation response

Although the inflammatory response is a natural defense mechanism that could be initiated by tissue injury [22], it can be beneficial or deleterious. After calving, the inflammatory and immune response in the endometrium attempts to eliminate any pathogenic bacterial contamination as well as initiate tissue repair as part of the involution process [45]. However, prolonged inflammation and cytokine production within the reproductive tract impair immune status and reproductive performance [45]. Therefore, the upregulation of genes related with the pro-inflammatory cascade (*TLR2, TLR4, NFKB1, TNF, IL6* and *IL1B*) in response to AAC at +14 d compared with +30 d indicated that pathogen elimination and tissue remodeling processes occurred earlier than in INO cows. In addition, the concentrations of blood biomarkers of inflammation [47] in these cows indicated a lower systemic inflammation status in AAC than INO cows.

### Oxidative stress response

Essential trace minerals such as Zn and Cu play a central role in metabolism and have the potential to reduce oxidative stress through several mechanisms. Evidence from human studies suggest that Zn is essential for expression and function of the transcription factor NFE2L2 [48]. This transcription factor helps control oxidative damage through its control of some antioxidant defense systems such as SOD1 activity [49] which requires Zn and Cu as co-factors. Therefore, the overall greater expression of *NFE2L2* and *SOD1* indicated that feeding AAC reduced oxidative stress within the endometrium and potentially help alleviate an overt inflammatory response. It is noteworthy that upregulation of *NFE2L2* also occurred in the PMNL and hoof corium (unpublished results) in the cows fed AAC, which strongly indicates a consistent effect of trace minerals on cellular stress through this transcription regulator.

### Eicosanoid metabolism

Among several biological functions, prostaglandins play a central role in the generation of an inflammatory response. They have pro-inflammatory properties and are responsible for typical signs of inflammation including redness, swelling and pain [50]. The synthesis of prostaglandins is partly dependent on Zn, hence, this trace mineral could play an indirect role in regulating enzymes involved in the arachidonic acid cascade that result in production of prostaglandins [51, 52]. Therefore, the upregulation of *PTGS2* and *PTGES* in cows fed AAC indicated that supplemental complexed Zn was more bioavailable for prostaglandin synthesis.

### Antimicrobial peptides

The upregulation of *MUC1*, a transmembrane glycoprotein abundantly expressed at the surface of the uterine epithelial tissue, in AAC cows indicated a greater ability to eliminate invading pathogenic bacteria [53]. This idea is supported by previous work demonstrating that *MUC1-*null mice were susceptible to chronic infections and inflammation, and had a markedly reduced fertility [54].

## Conclusions

Taken together, our findings reveal that supplementation with Zn, Mn, and Cu from AA complexes and Co from Co glucoheptonate during the transition period improved PMNL function and likely confer these cells a greater capacity to control invading pathogens. The robust inflammatory response coupled with the anti-oxidant response discerned from the transcriptome data in the uterine samples of cows fed complexed trace minerals likely allowed for a faster uterine recovery. These data indicate that the benefits of trace minerals from AA complexes extend beyond an improvement of liver function and productive performance [10]. Although our findings suggest that peripheral and uterine immune function was improved in cows supplemented with more bioavailable forms of trace minerals, further research to evaluate the clinical impact of that supplementation is warranted. Such research also will help to better define complexed trace mineral requirements beyond productive purposes.

## Additional files

Additional file 1: PMNL Isolation: Blood (120 mL), collected in ACD solution A vacutainer tubes, was mixed well by inversion and placed on ice until PMN isolation (within ~30 min). Tubes were combined into three 50-mL conical tubes (Fisher Scientific, Pittsburgh, PA) and centrifuged at 918 × g for 30 min at 4 °C. The plasma, buffy coat, and approximately one-third of the red blood cells (RBC) were removed and discarded. Cells were lysed with 25 mL of deionized water at 4 °C, homogenized gently by inversion, and then 5 mL of 5 × PBS (pH 7.4) at 4 °C was added, in order to restore an iso-osmotic environment. The cell suspension was centrifuged at 330 × g for 10 min at 4 °C and the supernatants were decanted. Ten milliliters of 1 × PBS at 4 °C was added in each tube, homogenized until there was nothing attached to the bottom of the tube, and then the three tubes were combined in one. The cell suspension was centrifuged at 663 × g for 5 min at 4 °C and the supernatants were discarded. The remaining RBC were lysed with 8 mL of deionized water at 4 °C, homogenized gently by inversion and 2 mL of 5 × PBS at 4 °C was added. The samples were centrifuged at 663 × g for 5 min at 4 °C and the supernatant was discarded. Two subsequent washings using 10 mL of 1 × PBS at 4 °C were performed, centrifuged at 663 × g for 5 min at 4 °C and supernatant discarded. Prior to the last centrifugation, 100 μL of the cell suspension were aliquoted for further PMN concentration and cell viability analysis. (DOC 43 kb)

Additional file 2: RNA extraction: Approximately 40 mg of frozen tissue was weighed and immediately placed in ice-cold 1 mL Qiazol reagent (Qiagen 75842; Qiagen Inc., Valencia, CA) for homogenization. After homogenization, the samples were centrifuged for 10 min at 12,000 × g at 4 °C to remove the insoluble material. The supernatant was transferred to a collection tube and incubated for 5 min on ice. Chloroform (200 µL) was added to each tube and the sample incubated at room temperature for 3 min. Subsequently, samples were centrifuged for 15 min at 12,000 × g at 4 °C, and the upper phase was transferred to a new collection tube without disturbing the mid and lower phases. A second wash was performed with 100% ethanol; 750 µL was added and transferred to a miRNeasy Mini Kit columns (Cat. No: 217004, Qiagen). Genomic DNA was removed on column from RNA samples with RNase-free DNase I, using the recommended protocol provided with the miRNeasy Mini Kit. RNA concentration was measured with a NanoDrop ND-1000 spectrophotometer (Thermo Fischer Scientific; Wilmington, DE), while the RNA quality was assessed using the Agilent 2100 Bioanalyzer system (Agilent Technologies, Santa Clara, CA). Samples of RNA used for analysis had an RNA integrity number ≥7.0. (DOC 44 kb)

Additional file 3: Function of the genes measured in the PMNL. (DOC 67 kb)

Additional file 4: Function of the genes measured in the endometrium. (DOC 61 kb)

Additional file 5: Features of used primers for qPCR analysis. Hybridization position, sequence, and amplicon size of primers for Bos taurus used to analyze gene expression. (DOC 107 kb)

Additional file 6: Sequencing results of PCR products from primers of genes used for this experiment. (DOC 62 kb)

Additional file 7: qPCR performance among the genes measured in PMNL. (DOC 77 kb)

Additional file 8: qPCR performance among the genes measured in the endometrium tissue. (DOC 71 kb)

## Abbreviations

AAC: Amino acid-complexed Zn, Mn, Cu, and Co
ACD: Citric acid and dextrose
DIM: Days in milk
DMI: Dry matter intake
IACUC: Institutional Animal Care and Use Committee
INO: Inorganic trace mineral mix
PMNL: Polymorphonuclear leukocyte
PPAR: Peroxisome proliferator-activated receptors
ROM: Reactive oxygen metabolites
