# Supplementary material for: Peripheral leukocyte and endometrium molecular biomarkers of inflammation and oxidative stress are altered in peripartal dairy cows supplemented with Zn, Mn, and Cu from amino acid complexes and Co from Co glucoheptonate
Source: J Anim Sci Biotechnol. 2017 May 1;8:33. doi: 10.1186/s40104-017-0163-7 (PMC5410708; doi:10.1186/s40104-017-0163-7)
Supplement: Supplementary file 1 — PMNL Isolation: Blood (120 mL), collected in ACD solution A vacutainer tubes, was mixed well by inversion and placed on ice until PMN isolation (within ~30 min). Tubes were combined into three 50-mL conical tubes (Fisher Scientific, Pittsburgh, PA) and centrifuged at 918 × g for 30 min at 4 °C. The plasma, buffy coat, and approximately one-third of the red blood cells (RBC) were removed and discarded. Cells were lysed with 25 mL of deionized water at 4 °C, homogenized gently by inversion, and then 5 mL of 5 × PBS (pH 7.4) at 4 °C was added, in order to restore an iso-osmotic environment. The cell suspension was centrifuged at 330 × g for 10 min at 4 °C and the supernatants were decanted. Ten milliliters of 1 × PBS at 4 °C was added in each tube, homogenized until there was nothing attached to the bottom of the tube, and then the three tubes were combined in one. The cell suspension was centrifuged at 663 × g for 5 min at 4 °C and the supernatants were discarded. The remaining RBC were lysed with 8 mL of deionized water at 4 °C, homogenized gently by inversion and 2 mL of 5 × PBS at 4 °C was added. The samples were centrifuged at 663 × g for 5 min at 4 °C and the supernatant was discarded. Two subsequent washings using 10 mL of 1 × PBS at 4 °C were performed, centrifuged at 663 × g for 5 min at 4 °C and supernatant discarded. Prior to the last centrifugation, 100 μL of the cell suspension were aliquoted for further PMN concentration and cell viability analysis. (DOC 43 kb) [file 40104_2017_163_MOESM1_ESM.doc]

**Additional File 1.**

**PMNL Isolation:** Blood (120 mL), collected in ACD solution A vacutainer tubes, was mixed well by inversion and placed on ice until PMN isolation (within ~30 min). Tubes were combined into three 50-mL conical tubes (Fisher Scientific, Pittsburgh, PA) and centrifuged at 918 × g for 30 min at 4°C. The plasma, buffy coat, and approximately one-third of the red blood cells (**RBC**) were removed and discarded. Cells were lysed with 25 mL of deionized water at 4°C, homogenized gently by inversion, and then 5 mL of 5 × PBS (pH 7.4) at 4°C was added, in order to restore an iso-osmotic environment. The cell suspension was centrifuged at 330 × g for 10 min at 4°C and the supernatants were decanted. Ten milliliters of 1 × PBS at 4°C was added in each tube, homogenized until there was nothing attached to the bottom of the tube, and then the three tubes were combined in one. The cell suspension was centrifuged at 663 × g for 5 min at 4°C and the supernatants were discarded. The remaining RBC were lysed with 8 mL of deionized water at 4°C, homogenized gently by inversion and 2 mL of 5 × PBS at 4°C was added. The samples were centrifuged at 663 × g for 5 min at 4°C and the supernatant was discarded. Two subsequent washings using 10 mL of 1 × PBS at 4°C were performed, centrifuged at 663 × g for 5 min at 4°C and supernatant discarded. Prior to the last centrifugation, 100 μL of the cell suspension were aliquoted for further PMN concentration and cell viability analysis.

The neutrophil pellet was homogenized with 1.5 mL of 1 × PBS at 4°C and transferred to a 2-mL RNase-DNase-free reinforced O-ring tube (Omni International, Kennesaw, GA) containing one stainless steel bead, 5 mm (Qiagen, Hilden, Germany), centrifuged at 1,435 × g for 5 min at 4°C, and then the supernatant was discarded. Immediately, 1 mL of Qiazol (Qiagen, Hilden, Germany) was added to the samples and homogenized twice in a Beadbeater (Biospec Products, Bartlesville, OK; catalog no. 607) for 30 s. During all the isolation process samples were kept on ice while outside of the centrifuge. The homogenized solution was transferred to a 2 mL DNase-RNase free tube (USA Scientific Inc., Ocala, FL) and stored in -80°C until further analysis. RNA concentration was measured with a NanoDrop ND-1000 spectrophotometer (Thermo Fischer Scientific; Wilmington, DE), while the RNA quality was assessed using the Agilent 2100 Bioanalyzer system (Agilent Technologies, Santa Clara, CA). Samples of RNA used for analysis had an RNA integrity number ≥7.0.

**PMNL Differential and Viability Analysis:** From the aliquot obtained during the PMN isolation process, 20 μL were transferred to a 5-mL falcon tube (Corning Incorporated, Durham, NC), added 150 μL of 1 × PBS at 4°C and 100 μL of a granulocyte primary antibody solution (CH138A, Veterinary Microbiology and Pathology, Washington State University, Pullm an, WA), homogenized by vortex and incubated on ice for 15 min. A washing step was performed three times by adding 1 mL of 1 × PBS at 4°C, homogenized by vortexing and centrifuged at 1,012 × *g* for 3 min at 4°C. The supernatant was aspirated using a glass transfer pipette (Fisher Scientific, Pittsburgh, PA) until ~100 μL remained at the bottom of the tube. Then, 50 μL of a second antibody solution (4 μg/mL in 1 × PBS) was added (Goat Anti-Mouse IgM, Human ads-PE; SouthernBiotech, Birmingham, AL) and 50 μL of propidium iodide solution (50 μg/mL in 1 × PBS; Sigma-Aldrich, St. Louis, MO), homogenized by vortex and incubated on ice for 15 min. Two washings were performed as described above. Cells were fixed with 150 μL of 4% paraformaldehyde (Sigma-Aldrich) and preserved at 4°C until flow cytometry reading (LSR II, Becton Dickinson, San Jose, CA).
