# Supplementary material for: Peripheral leukocyte and endometrium molecular biomarkers of inflammation and oxidative stress are altered in peripartal dairy cows supplemented with Zn, Mn, and Cu from amino acid complexes and Co from Co glucoheptonate
Source: J Anim Sci Biotechnol. 2017 May 1;8:33. doi: 10.1186/s40104-017-0163-7 (PMC5410708; doi:10.1186/s40104-017-0163-7)
Supplement: Supplementary file 3 — Function of the genes measured in the PMNL. (DOC 67 kb) [file 40104_2017_163_MOESM3_ESM.doc]

**Additional file 3.** Function of the genes measured in the PMNL.

| Inflammation | |
| --- | --- |
| *DDX58* | Viral RNA recognition and regulation of immune responses |
| *IPS1* | DNA recognition and regulation of immune responses |
| *NFKB1* | Transcription factor that is activated by cytokines, bacterial or viral products, etc. |
| *STAT3* | Transcription factor that mediates cellular responses to interleukins, and other growth factors |
| *TLR2* | Pathogen recognition and regulation of immune responses |
| *TLR4* | Pathogen recognition and regulation of immune responses |
| *TNF* | Cytokine that regulates inflammatory response |
| *ZBP1* | DNA recognition and regulation of immune responses |
| Oxidative stress | |
| *MPO* | Antimicrobial activity |
| *NFLE2L2* | Transcription factor that regulates genes that contain antioxidant response |
| *NOX1* | Generate superoxide or hydrogen peroxide |
| *S100A8* | Stimulate neutrophil chemotaxis and adhesion |
| *SOD1* | Present in the cytoplasm. Destroy superoxide radicals |
| *SOD2* | Present in the mitochondria. Destroy superoxide radicals |
| *SOD3* | Present in the extracellular space. Destroy superoxide radicals |
| Eicosanoids | |
| *ALOX5AP* | Leukotrienes synthesis |
| *LTA4H* | Leukotrienes synthesis |
| *PLA2G4A* | Release arachidonic acid from the membrane |
| *PTGS2* | Prostaglandin synthesis |
| Transcription factors | |
| *PPARA* | Transcription factor involved in endothelial cell inflammatory responses |
| *PPARD* | Transcription factor involved in endothelial cell inflammatory responses |
| *PPARG* | Transcription factor involved in endothelial cell inflammatory responses |
| *RXRA* | Transcription factor involved in endothelial cell inflammatory responses |
| Receptors | |
| *ADORA1* | G-protein coupled receptor that inhibits adenylyl cyclase |
| *ENTPD1* | Hydrolyze extracellular ATP and ADP to AMP |
| *IL10* | Cytokine with pleiotropic effects in immunoregulation and inflammation |
| *IL1B* | Cytokine that mediates inflammatory response |
| *ITGAM* | Neutrophils adherence and phagocytosis |
| *ITGB2* | Cell adhesion and signaling |
| *P2RY11* | G-protein coupled receptor |
| *PANX1* | Post-injury inflammatory response and tissue regeneration |
| *SELL* | Leukocytes migration |
| *TLN1* | Leukocytes adhesion |
| *VCL* | Cell adhesion |
| Glucose metabolism | |
| *LDHA* | Conversion of L-lactate and NAD to pyruvate and NADH in the final step of anaerobic glycolysis |
| *SLC2A1* | Glucose transporter |
