# Supplementary material for: Peripheral leukocyte and endometrium molecular biomarkers of inflammation and oxidative stress are altered in peripartal dairy cows supplemented with Zn, Mn, and Cu from amino acid complexes and Co from Co glucoheptonate
Source: J Anim Sci Biotechnol. 2017 May 1;8:33. doi: 10.1186/s40104-017-0163-7 (PMC5410708; doi:10.1186/s40104-017-0163-7)
Supplement: Supplementary file 4 — Function of the genes measured in the endometrium. (DOC 61 kb) [file 40104_2017_163_MOESM4_ESM.doc]

**Additional file 4.** Function of the genes measured in the endometrium.

| Inflammation | |
| --- | --- |
| *IL10* | Cytokine with pleiotropic effects in immunoregulation and inflammation |
| *IL1B* | Cytokine that mediates inflammatory response |
| *IL6* | Cytokine that mediates inflammation |
| *IL8* | Attracts neutrophils, basophils, and T-cells |
| *MYD88* | Signal transducer in the interleukin-1 and Toll-like receptor signaling pathways |
| *NFKB1* | Transcription factor that is activated by cytokines, bacterial or viral products, etc. |
| *SAA* | Recruit immune cell to inflammatory sites |
| *STAT3* | Transcription factor that mediates cellular responses to interleukins, and other growth factors |
| *TLR2* | Pathogen recognition and regulation of immune responses |
| *TLR4* | Pathogen recognition and regulation of immune responses |
| *TNF* | Cytokine that regulates inflammatory response |
| Oxidative stress | |
| *NFLE2L2* | Transcription factor that regulates genes that contain antioxidant response |
| *NOS3* | Synthesize nitric oxide which regulated leukocytes adhesion |
| *NRROS* | Limit reactive oxygen metabolites production by phagocytes during inflammatory response |
| *SOD1* | Present in the cytoplasm. Destroy superoxide radicals |
| *SOD2* | Present in the mitochondria. Destroy superoxide radicals |
| *SOD3* | Present in the extracellular space. Destroy superoxide radicals |
| Eicosanoids | |
| *ALOX5* | Leukotrienes synthesis |
| *ALOX5AP* | Leukotrienes synthesis |
| *LTA4H* | Leukotrienes synthesis |
| *LTC4S* | Leukotrienes synthesis |
| *PLA2G4A* | Release arachidonic acid from the membrane |
| *PTGDS* | Prostaglandins synthesis |
| *PTGES* | Prostaglandins synthesis |
| *PTGS2* | Prostaglandin synthesis |
| Transcription factors | |
| *PPARA* | Transcription factor involved in endothelial cell inflammatory responses |
| *PPARD* | Transcription factor involved in endothelial cell inflammatory responses |
| *PPARG* | Transcription factor involved in endothelial cell inflammatory responses |
| *RXRA* | Transcription factor involved in endothelial cell inflammatory responses |
| Antimicrobial peptides | |
| *MUC1* | Form an antimicrobial mucous barrier on epithelial surfaces |
