# Supplementary material for: Peripheral leukocyte and endometrium molecular biomarkers of inflammation and oxidative stress are altered in peripartal dairy cows supplemented with Zn, Mn, and Cu from amino acid complexes and Co from Co glucoheptonate
Source: J Anim Sci Biotechnol. 2017 May 1;8:33. doi: 10.1186/s40104-017-0163-7 (PMC5410708; doi:10.1186/s40104-017-0163-7)
Supplement: Supplementary file 5 — Features of used primers for qPCR analysis. Hybridization position, sequence, and amplicon size of primers for Bos taurus used to analyze gene expression. (DOC 107 kb) [file 40104_2017_163_MOESM5_ESM.doc]

**Additional file 5.** Features of used primers for qPCR analysis. Hybridization position, sequence, and amplicon size of primers for *Bos taurus* used to analyze gene expression.

| Symbol | | Primers^1^ | | Primers (5’-3’) | | bp^2^ |
| --- | --- | --- | --- | --- | --- | --- |
| *ADORA1* | F. 2024 | | CTGTGTTCCTGACCATGATGCT | | 100 | |
| *ADORA1* | R. 2123 | | ATTTCTGGACCCGTTCTTTCTG | |  | |
| *ALOX5* | F.500 | | TCATCAACCGCTTCATGCA | | 127 | |
| *ALOX5* | R.626 | | GTGATTCATGACCCGCTCAGA | |  | |
| *ALOX5AP* | F.114 | | ACAAGGTGGAGCACGAAAGC | | 100 | |
| *ALOX5AP* | R.213 | | ACACAGTTCTGGTTGGCAGTGT | |  | |
| *DDX58* | F.236 | | TCAGAAGCATGGGAAAAATCAG | | 102 | |
| *DDX58* | R.345 | | AGGAGGATGTGAGCATCTA | |  | |
| *ENTPD1* | F. 1439 | | ACATGCTGAACCTGACCAACAA | | 102 | |
| *ENTPD1* | R. 1540 | | GGATCAGGGAGAAGAGGACCAT | |  | |
| *IL10* | F.171 | | GAAGGACCAACTGCACAGCTT | | 98 | |
| *IL10* | R.268 | | AAAACTGGATCATTTCCGACAAG | |  | |
| *IL1B* | F. 30 | | ATTCTCTCCAGCCAACCTTCATT | | 100 | |
| *IL1B* | R. 129 | | TTCTCGTCACTGTAGTAAGCCATCA | |  | |
| *IL6* | F.190 | | CCAGAGAAAACCGAAGCTCTCAT | | 100 | |
| *IL6* | R.289 | | CCTTGCTGCTTTCACACTCATC | |  | |
| *IL8* | F.6 | | GACAGCAGAGCTCACAAGCATCT | | 105 | |
| *IL8* | R.110 | | AAGCTGCCAAGAGAGCAACAG | |  | |
| *IPS1* | F. 28 | | ACTCCGGCCTAACTCTCTCCTAA | | 96 | |
| *IPS1* | R.136 | | TGATGAGTTCATTGGCTTTGC | |  | |
| *ITGAM* | F.268 | | GGCTTGTCTCTTGCATTTGCT | | 95 | |
| *ITGAM* | R.362 | | CCATTTGCATAGGTGTTCTCCTT | |  | |
| *ITGB2* | F. 1231 | | GACACCCTGAAAGTCACCTACGA | | 108 | |
| *ITGB2* | R. 1338 | | GAAGGTGATCGGGACGTTGAT | |  | |
| *LDHA* | F.661 | | CAAGAGGTACCACTGCCCAT | | 104 | |
| *LDHA* | R.763 | | CACCTTGGCTAAAGGAACCA | |  | |
| *LTA4H* | F.1042 | | ACATTTGTGGACGACTGTTTGGT | | 98 | |
| *LTA4H* | R.1141 | | TGGGTCTCCCCAAAAGTCTTT | |  | |
| *LTC4S* | F.217 | | CATCTACCGAGCCCAAGTGAA | | 100 | |
| *LTC4S* | R.316 | | CAGTGCTGCCGCACCTT | |  | |
| *MPO* | F.1311 | | AGCCATGGTCCAGATCATCAC | | 105 | |
| *MPO* | R.1415 | | ACCGAGTCGTTGTAGGAGCAGTA | |  | |
| *MUC1* | F. 1198 | | CAGGAGCTGCAGAGAAGCATT | | 100 | |
| *MUC1* | R. 1297 | | CCACAGATCCTGGCCTGAAC | |  | |
| *MYD88* | F. 367 | | GGAGGACTGCCAAAAGTATATTCTG | | 105 | |
| *MYD88* | R. 471 | | GCCATGTCATTTATCCGAGTTATG | |  | |
| *NFKB1* | F.172 | | TTCAACCGGAGATGCCACTAC | | 95 | |
| *NFKB1* | R.266 | | ACACACGTAACGGAAACGAAATC | |  | |
| *NOS3* | F. | | GCCGGAACAGCACAAGAGTT | |  | |
| *NOS3* | R. | | GTGTTGCTGGACTCCTTTCTCTTC | |  | |
| *NOX1* | F. | | GTTTTACCGCTCCCAGCAGAA | |  | |
| *NOX1* | R. | | GGATGCCATTCCAGGAGAGAG | |  | |
| *NFE2L2* | F. | | TGGTTGCCTCTCACTACCCATTGT | |  | |
| *NFE2L2* | R. | | GCTTGTGCTGCCATCGAGTGATTT | |  | |
| *NRROS* | F. | | CTGCCTGGGTTTTCACTTTCTG | |  | |
| *NRROS* | R. | | AGCCACTCCATCCAGCAACT | |  | |
| *P2RY11* | F. 152 | | AGTGGCCTCCAAGATGACTTTC | | 103 | |
| *P2RY11* | R. 254 | | GCTCCCGGCTGCAGAAG | |  | |
| *PANX1* | F. 599 | | AGAAAGCCTCCAGACATTTAATCG | | 124 | |
| *PANX1* | R. 722 | | AACTCATCCGAGAGGGAGGAA | |  | |
| *PLA2G4A* | F.1134 | | CTCCATGTCAAACCCGATGTC | | 105 | |
| *PLA2G4A* | R.1238 | | GTCAGGCGCCATAAAAGTACCA | |  | |
| *PPARA* | F.729 | | CATAACGCGATTCGTTTTGGA | | 102 | |
| *PPARA* | R.830 | | CGCGGTTTCGGAATCTTCT | |  | |
| *PPARD* | F. 460 | | TGTGGCAGCCTCAATATGGA | | 100 | |
| *PPARD* | R. 559 | | GACGGAAGAAGCCCTTGCA | |  | |
| *PPARG* | F. 135 | | CCAAATATCGGTGGGAGTCG | | 101 | |
| *PPARG* | R. 235 | | ACAGCGAAGGGCTCACTCTC | |  | |
| *PTGDS* | F. | | CATTGTGTTCCTGCCGAAGA | |  | |
| *PTGDS* | R. | | AGAAGGGCCAGGTCCTGAGA | |  | |
| *PTGES* | F. | | FGGAACGACCCAGATGTGGAA | |  | |
| *PTGES* | R. | | GTCCGAGGAAAGAGTAGACAAAGC | |  | |
| *PTGS2* | F.1472 | | CGTTTTCTCGTGAAGCCCTATG | | 102 | |
| *PTGS2* | R.1573 | | CTCCATGGCATCTATGTCTCCAT | |  | |
| *RXRA* | F. 224 | | TGTCCCCGATGAGCTTGAAG | | 133 | |
| *RXRA* | R. 356 | | GAGGCGTACTGCAAACACAAGT | |  | |
| *S100A8* | F. 53 | | ACACCATGCTGACGGATCTG | | 100 | |
| *S100A8* | R. 152 | | TCCCTATAGACGGCGTGGTAA | |  | |
| *SAA3* | F. 50 | | GGGCATCATTTTCTGCTTCCT | | 106 | |
| *SAA3* | R. 155 | | TTGGTAAGCTCTCCACATGTCTTTAG | |  | |
| *SELL* | F.1506 | | AAGACCACGGGAAAAAAGGATT | | 105 | |
| *SELL* | R. 1610 | | ATGTCATCAGGTAGAGCATGGAATT | |  | |
| *SLC2A1* | F. 275 | | CCCCCAGAAGGTGATTGAAG | | 135 | |
| *SLC2A1* | R. 409 | | GAACCAATCATGCCTCCCAC | |  | |
| *SOD1* | F. 256 | | TGGAGATGCACAGATACACAGCTA | | 101 | |
| *SOD1* | R. 356 | | GCTGTCACATTGCCCAGGT | |  | |
| *SOD2* | F.620 | | TGTGGGAGCATGCTTATTACCTT | | 95 | |
| *SOD2* | R.714 | | GCATCCCTAGAAACTCTGGTCAA | |  | |
| *SOD3* | F. 106 | | CATGGATATCATCAAGCAAGACATG | | 108 | |
| *SOD3* | R. 238 | | GCTGGTCCTGGCAGGTAAACA | |  | |
| *STAT3* | F.3804 | | GGTAGCATGTGGGATGGTCTCT | | 110 | |
| *STAT3* | R.3913 | | GCATCCCTAGAAACTCTGGTCAA | |  | |
| *TLN1* | F.942 | | TTCCTGCCCAAGGAGTATGTG | | 100 | |
| *TLN1* | R.1041 | | AGCGTACCTTGGCCTCAATCT | |  | |
| *TLR2* | F.3182 | | CCATGTCTGGAGAGGGTGTT | | 102 | |
| *TLR2* | R.3283 | | GGGGACACAAAACAGCACTT | |  | |
| *TLR4* | F.102 | | GCTGTTTGACCAGTCTGATTGC | | 100 | |
| *TLR4* | R.203 | | GGGCTGAAGTAACAACAAGAGGAA | |  | |
| *TNF* | F. 174 | | CCAGAGGGAAGAGCAGTCCC | | 114 | |
| *TNF* | R. 287 | | TCGGCTACAACGTGGGCTAC | |  | |
| *VCL* | F.1778 | | CATCTCAGCTCCAAGACTCCTTAAA | | 103 | |
| *VCL* | R.1880 | | TTGATGGGAGTCGTGGTATCAC | |  | |
| *ZBP1* | R. 774 | | GTGATTCCTCTGCATCTTTTCATG | | 100 | |
| *ZBP1* | F. 875 | | CCCAGGAGACACAGACCTTGA | |  | |

^1^ Primer direction (F – forward; R – reverse) and hybridization position on the sequence.

^2^ Amplicon size in base pair (bp).
