# Supplementary material for: Peripheral leukocyte and endometrium molecular biomarkers of inflammation and oxidative stress are altered in peripartal dairy cows supplemented with Zn, Mn, and Cu from amino acid complexes and Co from Co glucoheptonate
Source: J Anim Sci Biotechnol. 2017 May 1;8:33. doi: 10.1186/s40104-017-0163-7 (PMC5410708; doi:10.1186/s40104-017-0163-7)
Supplement: Supplementary file 6 — Sequencing results of PCR products from primers of genes used for this experiment. (DOC 62 kb) [file 40104_2017_163_MOESM6_ESM.doc]

**Additional file 6.** Sequencing results of PCR products from primers of genes used for this experiment.

| Gene | Sequence |
| --- | --- |
| *ADORA1* | GGGGTCTGTCTCCTCGGATCAGGTTATACTTGTGAGAGGCAGAAAGAACGGGTCCAGAAATAACAGCT |
| *ALOX5* | CGCTTCATGCTAGGTTCCAGTCGTCCTGGAGCGACTTCCCGACTGCTTACTAAATCTTTGTCAGGCTAGGCAACACTATT |
| *ALOX5AP* | CGGGACTCCAGAGGACTGGGACGCTGGCCTTTGAGCGGGTCTACACTGCCAACCAGAACTGTGATAATTTT |
| *ENTPD1* | GCATGTAACAAGTTCACCCCTCCCCACTCCACCTATGTCTTCCGTCAGTGTCCTCTTCGTCCCGTGAATCCAGAACTAGG |
| *IL10* | GCACGAGTACTCTCGTCACTAGGAGTACCTTTAAGGGTTACCTGGGTGTGCCAAGGCCTTGTCCGGAAATGATCCAGTTTACGA |
| *IL1B* | ACAGCCATGGCACCGTACCTGAACCCATCAACGAAATGATCGGCTTACGTCACAGTGGACAGAGCACAATAGCACCCCC |
| *IL8* | CAGAGAAGCTAGTAAGCTCTCATTAAGCGCATGGTCGACAACGTATTGCAATGAGAAAGGAGGCTAGTGAGAAGAATGATGA |
| *ITGAM* | CGACTCTGCGTCGTGCGTGTGCCCACAGGTCACCAAATTTGCAAGGAGAACACCTATGCAAATGGAA |
| *ITGB2* | ATCGAATCGCAGGTGGACAGCCCAGAGGGACTGCGACGGCGTCCAGATCAACGTCCCGATCACCTTCAAGAGGG |
| *LDHA* | TCGCTTCCGTTTCTAGGTAGAGGGGGAGAGGCGCTATTACAGTACATGCCATGGGTGGATCCTTGGGGAGCATGGT |
| *MPO* | AGCCATGGGCTTAACATCACTTACCGGGATTACCTCGATATGGTGCTGGGGCGGGAGGCCCTAGAGGAAGTACCTGCGCGTA |
| *MUC1* | CAGATTTATAAACAGAGGGATTTTCTGGGCCTCTCAGAGATCAAGTTCAGGCCAGGATCTGTGGGA |
| *MYD88* | GACGCGGAGCATCGTAGAGGCCTTACGGTGGACTCTATAGACAGGCAGCATAACTCGGATAAATGGACATGGGCAACAC |
| *NFKB1* | CGATATCTTCGTGTCAAGCAAAAGTATTCGCAACACTGGAAGCACGAATGACAGATGCCTGTATACGGGGCATCAGAAGGCCGTA |
| *NOS2* | ACGAGGAAACGGGTGGAGGACTATTTCTTCCAGCTCAAGAACCAGAAGCGCTATCATG |
| *P2RY11* | GCTGTGATGTATCGGGTGCTGTGCGGCACAGCCTGGCCGTACCGCTTCTGCAGCCGGGAGCAATT |
| *PANX1* | GAAGGCGCGGGTGTCACTCAGCACGATACTGTTAGCCAGCCTCTACCTGGGCTATTACCTGAGCCTTTCCTCCCTCTCGGATGAGTTAAACCCCGGNAGCTCATGTACTTGACGATTAAATGTCGGGGGTTTTTA |
| *PON1* | GATAACTCATATCACGTCTGTCCACATCTGTGGGGGCCACAGTGATCTCATCTCAACAGTGAACGCTGACCCAA |
| *PPARA* | CGAGATCTGAAGCAAATTGAGGCAGAAATCCTTACGTGTGAGCATGACCTAGAAGATTCCGAAACCGCGA |
| *PPARD* | AGTCGTAACTGGGGGGACGATAAGCAGGGTTTCCCCAGTAAGCAGGGTGTCTTGACCCATGGCAGAGTTCGA |
| *PPARG* | ACAGCACGTGGGTCAGCGCTATGGGGAGCCCATCCCAGCGCCGCCACGCTGACCACGCATCTGGTCCCTGTCTGTGGCCATCTTCTCGGTGGGAGGCATGATTAGGTTCA |
| *RXRA* | GTCCAAAAACCGGTGGGCCAAAAGATGAAGAGAGGCATGTTGGAGACCTGGGCAATGTGACAGCT |
| *S100A8* | ATTGACGTCTACCACAAGTACTCCCTGAAAAAAGGGAATTACCACGCCGTCTATAGGGAAA |
| *SELL* | AGTAAGTCACCGGTAAATTTCATTCTCTCTCTCTCTGTATTCGTGGCTTCCAGCTTCAATTCCATGGCTCTACCTGGATGACATATAA |
| *SLC2A1* | ACAGCACGTGGGTCAGCGCTATGGGGAGCCCATCCCAGCGCCGCCACGCTGACCACGCATCTGGTCCCTGTCTGTGGCCATCTTCTCGGTGGGAGGCATGATTAGGTTCA |
| *SOD1* | GTCCAAAAACCGGTGGGCCAAAAGATGAAGAGAGGCATGTTGGAGACCTGGGCAATGTGACAGCT |
| *SOD2* | GCATGTTTGGCCGATTATCTGAGGCCATTTTGGAATGTGATCAACTGGGAGAATGTAACTGCAATAC |
| *STAT3* | GCATCCCTCTACGAGCACGGCTAGATGTGGTCGGCTACAGCCATCTTGTCTCAGTTGACCAGAGTTTCTAGGGATGCAA |
| *TLR2* | TTAAAAGAGTCACAATAGAAAGCTTAAGGTTTTTCTGGTTCCTTGTTTCACAACATTTAAATTCGGCGTTAGAATATTTGGATCGTCATAAAAACTTA |
| *TLR4* | GCATCCCTCACCGTTATGGTCAGGTGAATTCCTGGGATAAGGCCAGGCTTCCTCTTGTTGGTTACTTCAGCCAGAAA |
| *TNF* | TCACTCTCCGGGGCAGCTCCGGTGGTGGGACTCGTATGCCAATGCCCTCAT GGAA |
| *VCL* | GCAGTAGACCGGAGTCGGGAGGCAGTACCTCAGGAGGTGTCAGATGTTTTCAGTGATACCACGACTCCCATCAACACTTT |
