# Supplementary material for: Peripheral leukocyte and endometrium molecular biomarkers of inflammation and oxidative stress are altered in peripartal dairy cows supplemented with Zn, Mn, and Cu from amino acid complexes and Co from Co glucoheptonate
Source: J Anim Sci Biotechnol. 2017 May 1;8:33. doi: 10.1186/s40104-017-0163-7 (PMC5410708; doi:10.1186/s40104-017-0163-7)
Supplement: Supplementary file 7 — qPCR performance among the genes measured in PMNL. (DOC 77 kb) [file 40104_2017_163_MOESM7_ESM.doc]

**Additional file 7.** qPCR performance among the genes measured in PMNL.

| Gene | Median Ct^1^ | Median ∆Ct^2^ | Slope^3^ | (R^2^)^4^ | Efficiency^5^ | Relative mRNA abundance^6^ | 1/E∆Ct^7^ |
| --- | --- | --- | --- | --- | --- | --- | --- |
| *ITGB2* | 17.36 | -6.91 | -2.909 | 0.996 | 2.20 | 355.73 | 56.70 |
| *TLN1* | 18.51 | -5.70 | -3.070 | 0.996 | 2.12 | 72.13 | 11.50 |
| *IL1B* | 18.49 | -5.63 | -3.142 | 0.997 | 2.08 | 61.99 | 9.881 |
| *S100A8* | 19.20 | -4.88 | -3.180 | 0.996 | 2.06 | 34.27 | 5.463 |
| *SELL* | 19.60 | -4.53 | -3.125 | 0.997 | 2.09 | 28.08 | 4.476 |
| *SOD2* | 20.05 | -4.18 | -3.374 | 0.992 | 1.98 | 17.28 | 2.754 |
| *TLR2* | 21.60 | -2.63 | -2.986 | 0.993 | 2.16 | 9.50 | 1.514 |
| *SOD1* | 21.79 | -2.45 | -2.919 | 0.964 | 2.20 | 7.99 | 1.273 |
| *NFE2L2* | 21.64 | -2.56 | -2.910 | 0.992 | 2.20 | 7.62 | 1.215 |
| *RXRA* | 22.15 | -1.97 | -3.075 | 0.999 | 2.11 | 4.37 | 0.697 |
| *IPS1* | 22.67 | -1.63 | -2.945 | 0.994 | 2.18 | 4.15 | 0.661 |
| *ITGAM* | 22.34 | -1.92 | -3.204 | 0.998 | 2.05 | 3.97 | 0.633 |
| *PPARD* | 22.41 | -1.74 | -3.075 | 0.996 | 2.11 | 3.68 | 0.586 |
| *STAT3* | 22.56 | -1.59 | -3.213 | 0.997 | 2.05 | 3.12 | 0.498 |
| *LDHA* | 22.85 | -1.27 | -3.096 | 0.997 | 2.10 | 2.57 | 0.409 |
| *NFKB1* | 23.17 | -1.05 | -3.195 | 0.994 | 2.06 | 2.13 | 0.339 |
| *ALOX5AP* | 23.12 | -1.03 | -3.204 | 0.996 | 2.05 | 2.10 | 0.335 |
| *VCL* | 23.50 | -0.68 | -3.230 | 0.995 | 2.04 | 1.62 | 0.259 |
| *DDX58* | 24.52 | 0.05 | -3.297 | 0.987 | 2.01 | 0.96 | 0.153 |
| *TLR4* | 24.21 | 0.08 | -3.212 | 0.992 | 2.05 | 0.95 | 0.151 |
| *ZBP1* | 24.38 | 0.21 | -2.919 | 0.994 | 2.20 | 0.84 | 0.134 |
| *TNF* | 24.60 | 0.32 | -3.755 | 0.967 | 1.85 | 0.82 | 0.131 |
| *LTA4H* | 25.21 | 0.94 | -3.200 | 0.992 | 2.05 | 0.51 | 0.081 |
| *ENTPD1* | 26.24 | 1.84 | -3.287 | 0.990 | 2.01 | 0.28 | 0.044 |
| *ADORA1* | 26.57 | 2.54 | -3.591 | 0.986 | 1.90 | 0.20 | 0.031 |
| *PANX1* | 27.15 | 2.93 | -3.123 | 0.993 | 2.09 | 0.12 | 0.018 |
| *PLA2G4A* | 27.70 | 3.35 | -3.066 | 0.992 | 2.12 | 0.08 | 0.013 |
| *PPARA* | 27.42 | 3.21 | -2.903 | 0.977 | 2.20 | 0.08 | 0.012 |
| *SLC2A1* | 28.36 | 3.98 | -3.421 | 0.986 | 1.96 | 0.07 | 0.011 |
| *NOX1* | 27.72 | 3.47 | -2.955 | 0.944 | 2.18 | 0.07 | 0.011 |
| *MPO* | 28.60 | 4.18 | -3.106 | 0.990 | 2.10 | 0.05 | 0.007 |
| *IL10* | 29.24 | 4.99 | -2.946 | 0.983 | 2.18 | 0.02 | 0.003 |
| *P2RY11* | 28.16 | 3.93 | -3.302 | 0.980 | 2.01 | 0.02 | 0.003 |
| *PPARG* | 28.67 | 4.19 | -2.909 | 0.978 | 2.20 | 0.01 | 0.001 |
| *PTGS2* | 30.38 | 6.06 | -3.045 | 0.948 | 2.13 | 0.00 | 0.000 |

^1^The median is calculated considering all time points and all cows.

^2^The median of ΔCt is calculated as [Ct gene – geometrical mean of Ct internal controls] for each time point and each cow.

^3^Slope of the standard curve.

^4^R2 stands for the coefficient of determination of the standard curve.

^5^Efficiency calculated as [10^(−1/slope)^].

^6^Relative mRNA abundance = 1/Efficiency ^Median Ct^

^7^1/E∆Ct = relative mRNA abundance/Σ relative mRNA abundance × 100.
